# Kidney Disease in Systemic Amyloidosis: A Review of Amyloid, Amyloid Serum A Protein, Leukocyte Chemotactic Factor 2, and Transthyretin Amyloid

**DOI:** 10.34067/KID.0000000600

**Published:** 2024-10-02

**Authors:** Raad Chowdhury, Sujal Shah, Sheron Latcha, Luisa Lobato

**Affiliations:** 1 Division of Kidney Medicine, Brigham and Women's Hospital, Boston, Massachusetts; 2 Department of Medical Oncology, Dana Farber Cancer Institute, Boston, Massachusetts; 3 Harvard Medical School, Boston, Massachusetts; 4 Department of Pathology, Brigham and Women's Hospital, Boston, Massachusetts; 5 Memorial Sloan Kettering Cancer Center, New york, NY; 6 Department of Nephrology, Centro Hospitalar Universitário de Santo António (CHUdSA), Porto, Portugal; 7 UMIB - Unit for Multidisciplinary Research in Biomedicine, ICBAS - School of Medicine and Biomedical Sciences, University of Porto, Porto, Portugal; 8 ITR - Laboratory for Integrative and Translational Research in Population Health, University of Porto, Porto, Portugal

**Keywords:** glomerular disease, onconephrology

## Abstract

Systemic amyloidoses are a group of disorders that can be hereditary or acquired and have various renal manifestations and outcomes. Light chain amyloid has been considered the most common renal amyloid and, thus, has been the focus of substantial research and therapeutic interest but with improvement in diagnostic techniques. However, there has been growing interest in rarer forms of renal amyloid, including amyloid serum A protein, leukocyte chemotactic factor 2 amyloid, and transthyretin amyloid. In this review, we provide an update on diagnostics, renal outcomes, and therapeutic landscape in these specific types of amyloid.

## Visual Abstract

**Figure:**
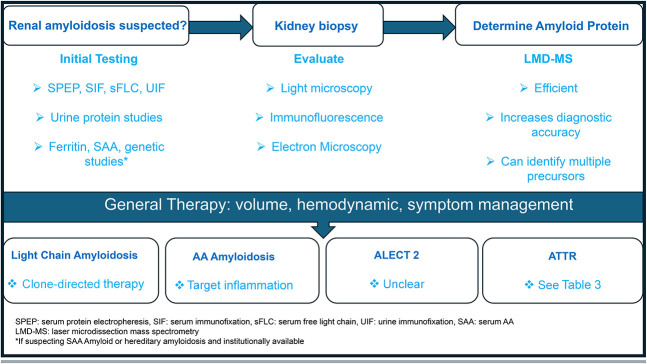

## Introduction

Amyloidoses are a multisystem disease caused by acquired or hereditary disorders and are characterized by the production of precursor protein that undergoes misfolding, leading to insoluble fibril formation and tissue deposition. The prevalence of renal amyloidosis in the largest series of native kidney biopsies is 1.6%, with light chain (LC) amyloid (AL) identified in 81%–86% of cases, amyloid serum A protein (AA) in 7%, and leukocyte chemotactic factor 2 (ALECT2) in 2.5%–2.7%.^1^ Transthyretin amyloid (ATTR), which predominantly affects the nerves and heart, can infrequently involve kidneys. Renal syndromes vary from nephrotic syndrome in AL to a more subclinical course with progressive CKD in ALECT2, whereas AA and ATTR have more varied renal courses.^1,33,40^

## Diagnostic Approach

Currently, over 40 amyloidogenic proteins have been identified, of which approxiamtely 15 cause kidney disease.^2,5^ The International Society of Amyloidosis (ISA) has proposed nomenclature for the type of systemic amyloid and the corresponding amyloid precursor protein (Table 1). The ISA recommends a low threshold for obtaining a tissue biopsy where amyloidosis is suspected because of the (*1*) rarity of these diseases, (*2*) substantial variability in their clinical manifestations, (*3*) evolution of impactful therapeutic options, (*4*) need for targeted therapy specific to each amyloid protein, and (*5*) dependence of therapeutic efficacy on disease stage at diagnosis and treatment.^3^ The ISA states that it is reasonable to forego the renal biopsy in a patient with cardiomyopathy, grade 2–3 myocardial radiotracer uptake on bone scintigraphy, and without evidence of a monoclonal gammopathy on serum and urine immunofixation (SIF, UIF) and serum-free LC (sFLC) testing. Initial noninvasive techniques to detect renal AL include SIF, UIF, serum and urine electrophoresis, and sFLC. Combining the SIF, UIF, and sFLC has a >98% sensitivity for detecting and identifying the isotype.^5^ However, genetic testing, imaging, and mass spectrometry are often required for the other types of amyloid. With respect to imaging, there are currently no US Food and Drug Administration–approved modalities.^2,3^ I-labeled serum amyloid protein scintigraphy binds all types of amyloid and can be used to diagnose the presence of disease and monitor response to treatment but cannot clarify the type of amyloid. Moreover, this method is only available in the United Kingdom and the Netherlands. ^99m^Tc-aprotinin scintigraphy is not suitable for assessment of renal amyloid because it is excreted through kidneys. ^18^F-florbetapir detects several types of amyloid but has a low affinity for ATTR and does not accurately detect renal amyloid. A recent, small, promising, clinical study demonstrated that ^124^I-p5+14 has panamyloid reactivity and is able to detect multiorgan amyloidosis.^6^

**Table 1:** Amyloid fibril proteins reported with kidney disease

Fibril Protein	Precursor Protein	S or L	A or H
AL	Immunoglobulin LC	S, L	A, H
AH	Immunoglobulin heavy chain	S, L	A
AA	Apo serum amyloid A	S	A, H
ATTR	Transthyretin, wild type	S	A
	Transthyretin, variants	S	H
AApoAI	Apolipoprotein A I, variants	S	H
AApoAII	Apolipoprotein A II, variants	S	H
AApoAIV	Apolipoprotein A IV, wild type	S	A
	Apolipoprotein A IV, variants	s	H
AApoCII	Apolipoprotein C II, variants	S	H
AApoCIII	Apolipoprotein C III, variants	S	H
AGel	Gelsolin, variants	S	H
ALys	Lysozyme, variants	S	H
ALECT2	Leukocyte chemotactic factor-2	S	A
AFib	Fibrinogen *α*, variants	S	H
ACal	Procalcitonin	S	A

A, acquired; AA, amyloid serum A protein; AFib, fibrinogen Aα-chain; AL, amyloid; ATTR, transthyretin amyloid; H, hereditary; L, localized; LC, light chain; S, systemic.

Because systemic amyloidosis is characterized by protein deposition in many different tissues, it is reasonable to biopsy sites that are more easily accessible and present a relatively lower risk of biopsy-related complications. For renal amyloidosis, renal biopsy provides both greater diagnostic accuracy and prognostic information and should be performed whenever feasible.^2^

Common extrarenal sites of involvement include the abdominal fat pad, rectum, salivary gland, and bone marrow. Fat pad biopsy with Congo red (CR) stain and polarized microscopy shows a sensitivity of 80% and specificity of 100% for identifying amyloid protein. Fine-needle aspiration with a 16-gauge needle is preferred to a 22-gauge needle because the latter may not yield adequate tissue to evaluate for some types of amyloid, such as ATTR, for which connective tissue is needed.^7^ If renal biopsy is contraindicated or cannot be obtained, evidence of AL in a fat pad, lip, or bone marrow biopsy and presence of proteinuria >500 mg/d is sufficient for diagnosis.^8–10^

Kidney biopsy specimens can be evaluated using light microscopy with CR staining, immunoassays, electron microscopy (EM), and laser microdissection mass spectrometry (LMD-MS). The first step in evaluation for amyloid is CR stain, which stains amyloid salmon pink and shows a pathognomonic apple-green birefringence when observed under polarized light. Even minor amyloid deposits can be detected with this technique, but expertise in preparing and interpreting the slides is paramount. The sensitivity of CR is 98%, but the absence of staining does not rule out amyloidosis.^7^ Thioflavin T or S stains are more sensitive than CR with polarization and easier to perform, but are used predominantly in the research setting.^10^

After establishing the presence of amyloid, the precursor protein must be identified. Antibody-based detection methods used to identify amyloid precursor proteins include immunohistochemistry (IHC), immunofluorescence (IF), and immune gold applied to frozen tissue on paraffin sections. For AL, commercially available antibodies for IF do not detect the mutated LC in 10%–15% of patients because of conformational changes and/or fragmentation of the LC, which mask specific epitopes targeted by the antibodies. IF and IHC techniques can additionally occasionally result in nonspecific trapping of circulating plasma proteins, resulting in false-positive results for AL.^11^ Although there are available antibodies that can reliably type precursor proteins for some types of hereditary amyloid (serum AA [SAA], ATTR, fibrinogen Aα-chain), there are currently no available antibodies to certain forms of hereditary amyloid.^11^ When the CR stain is equivocal, EM can demonstrate presence of amyloid, characterized by nonbranching, randomly arranged fibrils measuring 7–12 nm in diameter. Immuno-EM (IEM), which combines IHC and EM using gold-labeled antibodies that colocalize to protein within the amyloid fibrils and reduce background staining, can also be used to type amyloid protein in equivocal cases. IEM sensitivity is comparable with CR, but IEM has superior specificity (100% versus 80%).^14^

LMD-MS has emerged as the gold standard for determining the amyloid precursor protein. Sections of CR-positive tissue are microdissected, dissolved, and cleaved using trypsin, and the protein constituents are identified by comparison with a large database of known proteomes. A major advantage of mass spectrometry is that it identifies hundreds of proteins simultaneously, whereas only a handful of proteins can be identified by IF and IHC and, additionally, must be tested individually. LMD-MS has reduced the percentage of patients in whom amyloid cannot be typed.^12^ In one study, LMD-MS successfully identified the amyloid subtype in 98% of patients, compared with 43% for IHC.^13^ LMD-MS is additionally valuable in rare cases where more than one type of amyloid is present. It can also detect known amino acid substitutions in hereditary amyloidosis and has, thus, become an indispensable diagnostic tool for this disease. Because therapeutic efficacy is largely dependent on the stage of disease at the stime of treatment, there is ongoing research to develop noninvasive methods to enable earlier detection of renal amyloidosis.

## LC Amyloidosis

### Patient 1

**Figure 1:**
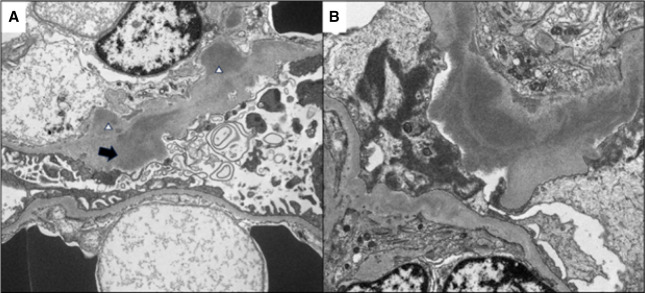
**Patient 1, EM of initial biopsy.** Received EM images from the patient's prior kidney biopsy at an outside institution revealed the presence of subepithelial (black arrow) and subendothelial (white arrowhead) deposits (A). The highest resolution EM images of these deposits precluded definite assessment of substructural appearance, limiting interpretation (B). In conjunction with the reported IF positivity for only IgA and C3 (not available for review), the possibility of IgA heavy chain disease was raised on review of the case. EM, electron microscopy; IF, immunofluorescence.

**Figure 2:**
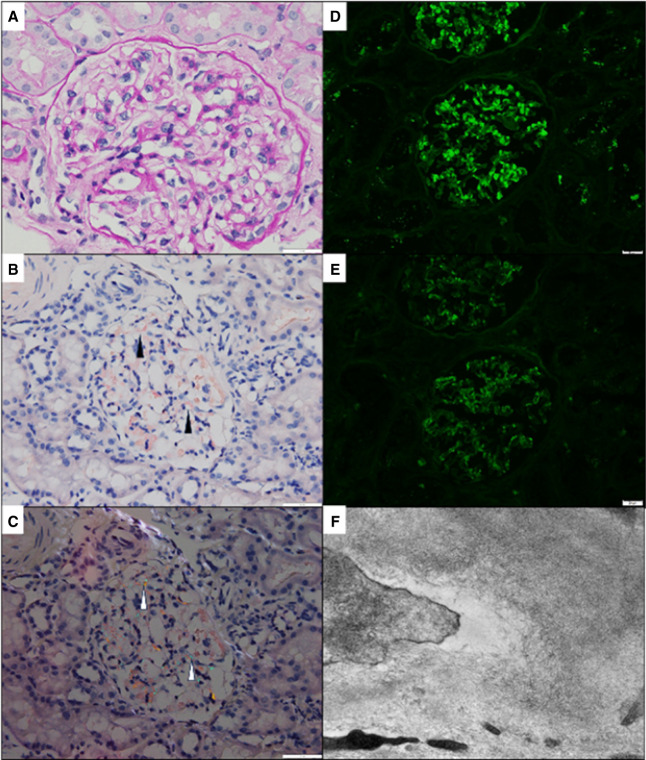
**AL (lambda)-type amyloidosis.** A repeat kidney biopsy was performed to further evaluate the patient's kidney disease. Light microscopic evaluation revealed glomeruli with only minimal-to-mild mesangial matrix expansion (A, original magnification ×400). CR stain showed only very minimal mesangial Congophilic material (black arrowhead; B, original magnification ×400), which revealed apple-green birefringence under polarized light (white arrowhead; C, original magnification ×400). IF microscopy revealed intense mesangial staining for lambda light chains (D, original magnification ×200), with only dull background/nonspecific staining for kappa light chains (E, original magnification ×200). There was no significant glomerular reactivity for IgA or the other immunoglobulin heavy chains (not pictured). Ultrastructural examination revealed the presence of nonbranching, randomly arranged fibrils within the mesangium and capillary loops (F); the fibrils measured 9.52 nm in average diameter. CR, Congo red.

A 67-year-old woman with a recent diagnosis of IgA nephropathy on renal biopsy is evaluated for nephrotic-range proteinuria (5 g/24 hours, on a maximum dose of angiotensin receptor blockers). Creatinine is 0.9 mg/dl. Workup revealed elevated lambda-free LCs, and bone marrow biopsy showed 45% involvement by lambda-restricted plasma cells, without evidence of amyloid by CR stain. A repeat kidney biopsy revealed LC amyloidosis (Figures 1 and 2). There was no evidence of cardiac involvement. She was started on daratumumab, cyclophosphamide, bortezomib, and dexamethasone. She achieved a complete hematological remission (CR) but with minimal improvement in proteinuria.

AL amyloid is the most common type of renal amyloidosis, with substantial morbidity and mortality. In a cohort of 234 patients, 50% had renal involvement. Among these, 40% had nephrotic syndrome and 18% required renal replacement therapy, with median time to initiation 13.8 months and a survival of less than 1 year once initiated.^15,16^ With the advent of more precise therapy over the past two decades, survival and quality of life have improved.

Renal outcomes are predicated on the degree of proteinuria and eGFR, and staging is essential. For example, eGFR <50 ml/min per 1.73 m^2^ and proteinuria >5 g/24 hours are considered as stage III and have up to an 85% risk of requiring dialysis in 3 years.^4^ Mortality in AL is dictated by cardiac involvement. The Mayo 2012 system incorporates cardiac biomarkers and the difference between involved FLC and uninvolved FLC (dFLC).^17^

Treatment of AL has improved in the past two decades with clone-directed therapy. Overall survival has increased since 1997, at that time it was under 2 years with standard-of-care colchicine.^15^ In 2021, the landmark ANDROMEDA trial showed that addition of daratumumab to cyclophosphamide, bortezomib, and dexamethasone (CyBorD) improved hematologic CR and increased cardiac and renal response at 6 months. In short, there was faster and deeper response with longer survival-free progression, and this is currently the standard of care.^18^

Second-line therapies, including immunomodulators, such as thalidomide and lenalidomide, have shown efficacy, but tolerability is an issue. Enhanced clearance and dissolution of amyloidomas was demonstrated using *in vivo* experimental models with fibril-reactive chimeric monoclonal antibody 11-1F4, and a phase 1A/B study with 27 patients receiving CAEL-101 at escalating doses had promising cardiac response.^19^ A phase 3 study using birtamimab, which neutralize soluble misfolded FLC, did not reach statistical significance of its primary outcome.^20^ Further studies are needed to validate these varied findings.

## AA Amyloidosis

### Patient 2

**Figure 3:**
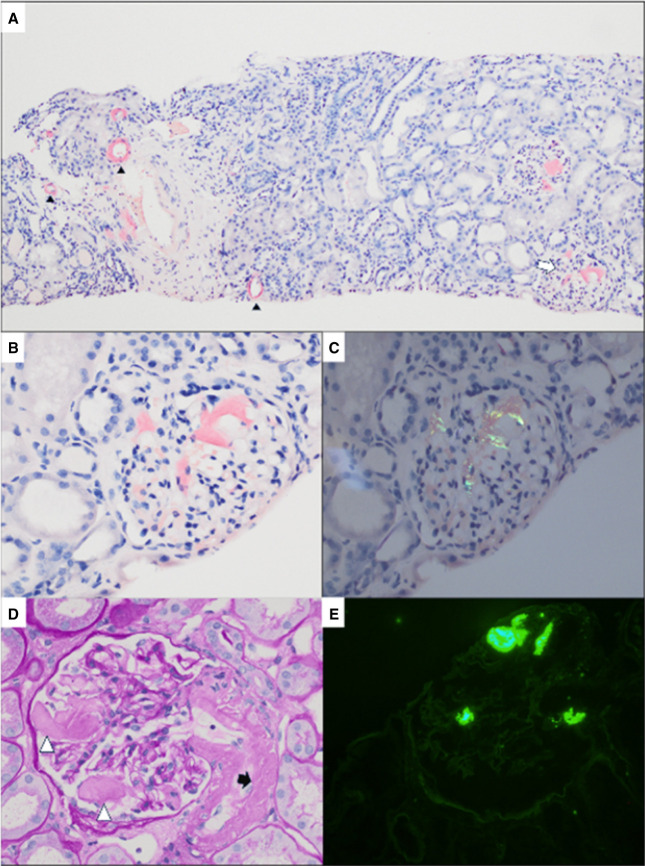
**AA amyloidosis.** Kidney biopsy demonstrated the presence of vascular (black arrowhead) and glomerular (white arrow) Congophilic material (A, original magnification ×100). Higher power examination of the glomerulus confirmed the presence of Congophilic material (B, original magnification ×400), which showed apple-green birefringence under polarized light (C, original magnification ×400). Light microscopy revealed mesangial expansion by amorphous, acellular, PAS-pale material (black arrow), with similar material also seen in the vessels (white arrowhead) by PAS stain (D, original magnification ×400). IF microscopy revealed mesangial staining for protein A (E, original magnification ×400), without concurrent positivity for immunoglobulin heavy or light chains or transthyretin (not pictured). EM revealed nonbranching, haphazardly arranged fibrils, which measured 10.85 nm in average diameter (not pictured). PAS, periodic acid–Schiff.

A 53-year-old woman with juvenile rheumatoid arthritis (RA) diagnosed at the age of 3 years was evaluated for an elevated creatinine of 2.16 mg/dl (baseline 0.6 mg/dl) and new-onset albuminuria of 2 g/d. The urinalysis was otherwise bland. A renal biopsy showed glomerular and vascular involvement with amyloid, with protein A immunoreactivity (Figure 3). After treatment with tofacitinib, inflammatory markers and serum creatinine improved.

AA was formerly the most common form of renal amyloidosis, but is now second to AL. In a review of 474 patients with renal amyloidosis, 33 (7.0%) were attributed to AA.^1^ The incidence in Western countries is believed to be decreasing to approximately one patient per million person-years with a concomitant increase in median age of diagnosis to 70 years, largely because of improvement in the control of inflammatory diseases.^21^ The incidence is still higher in resource-limited countries because of infections. The most common associations are inflammatory arthritis and RA, followed by infectious sequelae, such as in intravenous drug use.^22^ AA occurs because of hepatic oversynthesis of SAA through cytokine-mediated pathways, including IL-1, tumor necrosis factor-*α*, and IL-6.^23,24^ The amyloidogenic properties that lead to organ deposition are not entirely clear, but it is evident that the SAA burden is contributory.^22^

The prognosis of AA amyloidosis can be favorable provided SAA levels are suppressed by the control of inflammation. In a landmark study, mortality, amyloid burden, and renal prognosis were significantly worse in patients with high SAA levels. When median SAA concentrations were suppressed below 10 mg/L, there was regression of amyloid burden and improvement of survival.^22^ Unfortunately, SAA assays have not been standardized and are not routinely available.

Treatment is targeted toward the underlying inflammatory conditions and suppression of SAA levels. In RA, this involves use of disease-modifying antirheumatic drugs. In familial Mediterranean fever, a landmark study using colchicine showed reduced prevalence of nephropathy as compared with precolchicine studies.^25^ There has been experimental interest in interfering with amyloidogenic properties of AA. In one study, 89 of 183 patients were randomized to receive eprodisate, a low–molecular-weight sulfate that interferes with amyloid formation in murine models. In clinical trials, renal decline was attenuated in 24 of 89 patients in the intervention arm and 38 of 94 patients in the placebo arm. However, subsequent trials did not show similar benefits.^26^ The combination of miridesap, which decreases circulating SAA protein, along with the monoclonal antibody dezamizumab, which binds to formed amyloid fibrils and triggers macrophage-mediated removal, has not shown clear promise in AA.^27,28^ Regardless of the inflammatory trigger, suppressing its activity portends better outcomes.

## ALECT2 Amyloidosis

### Patient 3

**Figure 4:**
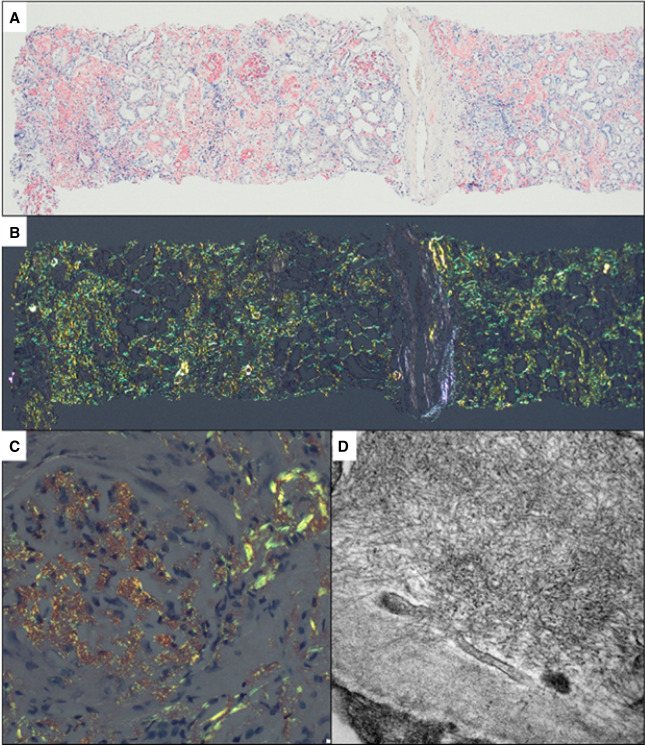
**ALECT2 amyloidosis.** Light microscopic evaluation of the kidney biopsy revealed extensive cortical interstitial, along with glomerular and vascular Congophilic material (A, original magnification ×40) throughout the entire core, with apple-green birefringence under polarized light (B, original magnification ×40). Higher power view of the glomeruli confirmed the presence of this amorphous, acellular, birefringent material in the mesangium, with occasional infiltration into the capillary loops, and in the vasculature (C, original magnification ×400). Ultrastructural examination showed the presence of subendothelial (D), mesangial, interstitial, and vascular nonbranching, irregularly arranged fibrils that measured 10.0 nm in average diameter. ALECT2, leukocyte chemotactic factor 2.

**Figure 5:**
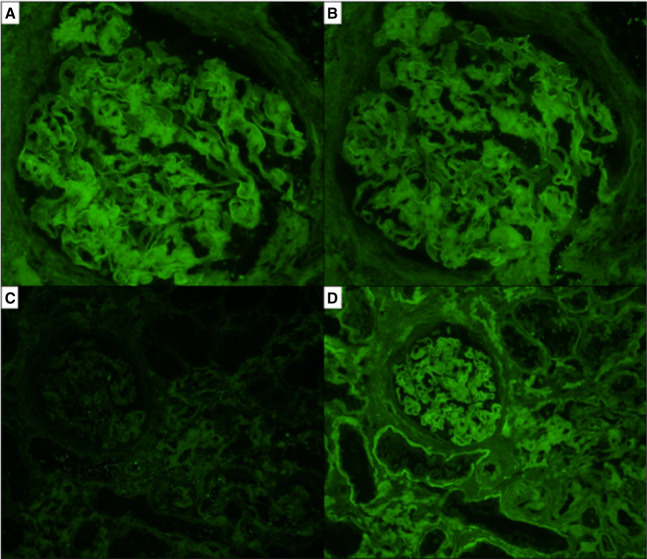
**Negative IF in ALECT2 amyloidosis.** IF microscopy revealed similar background, nonspecific reactivity for both kappa (A, original magnification ×400) and lambda (B, original magnification ×400) light chains, and the material was negative for the immunoglobulin heavy chains (not pictured). Further IF microscopy workup was negative for protein A (C, original magnification ×200) and revealed only nonspecific background staining for transthyretin (D, original magnification ×200).

A 71-year-old Hispanic man with a history of nephrolithiasis was evaluated for a gradual rise in creatinine. A kidney biopsy revealed extensive CR-positive fibrillary material (Figure 4), without IF reactivity for immunoglobulin heavy or LCs, protein A, or transthyretin (Figure 5), and mass spectrometry confirmed ALECT2 amyloidosis. At his 2-year follow-up, proteinuria has been stable, but serum creatinine has slowly increased.

In 2008, an idiopathic case of renal amyloidosis was attributed to leukocyte chemotactic factor 2-type amyloid (ALECT2) after biochemical fibril analysis.^29^ ALECT2 is believed to be produced in the liver, but the exact cause for aberrant stimulation is unclear. In a review of 72 patients with ALECT2, the mean age at diagnosis was 65 years and 92% of patients were Mexican. In this cohort, one third of patients progressed to ESKD, with serum creatinine the main predictor of progression. All patients were alive at roughly 2-year follow-up.^30^ This is in contrast to a previous study that reported 62% of patients with AL and 24% with AA surviving after a median follow-up of 25 months.^31^ This disparity likely results from the predominant kidney and liver deposition, without cardiac involvement. However, a case of cardiac involvement has been reported in a 72-year-old Hispanic patient.^32^ ALECT2 has also been reported in patients of Punjabi and Native American ethnicities and is potentially the second most common form of renal amyloid in Egypt.^33^ Currently, there are no treatment options.

## Transthyretin Amyloid Amyloidosis

### Patient 4

**Figure 6:**
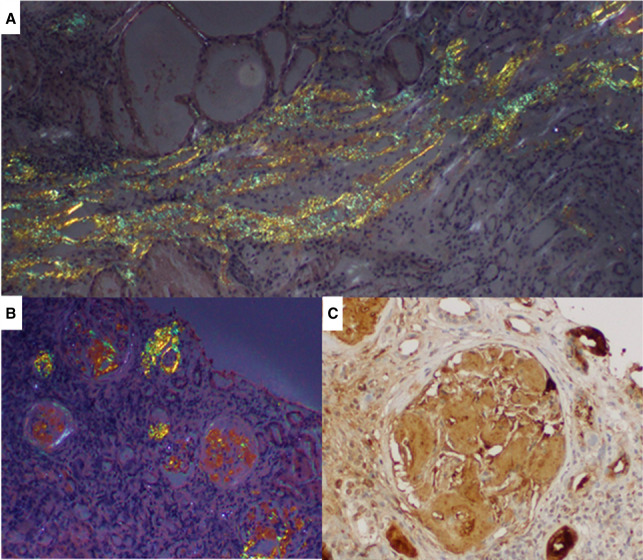
**ATTR amyloidosis (ATTRV30M).** Kidney biopsy exhibited extensive medullary interstitial deposits with CR affinity (not shown), presenting apple-green birefringence under polarized light (A, original magnification ×100). Prominent vascular pole Congophilic deposits, accompanied by involvement of mesangial areas, were observed with polarized light (B, original magnification ×20). The anti-TTR fixation, by the immunoperoxidase technique, demonstrated heavy glomerular positivity (C, original magnification ×200). ATTR, transthyretin amyloid.

A 68-year-old Portuguese woman with a long-standing history of hypertension and diabetes was referred for a urine albumin–creatinine ratio of 5568 mg/g with serum albumin 3.5 g/dl, serum creatinine 1.25 mg/dl, and eGFR CKD Epidemiology Collaboration creatinine–cystatin C 32 ml/min per 1.73 m^2^. The patient was orthostatic and edematous with lower extremity neuropathy. Family history was unremarkable. Kidney biopsy revealed amyloid involving the glomeruli, vascular poles, and medulla, with sparing of the cortical interstitium, characteristic of ATTR amyloid. ATTR IHC using anti-ATTR antibody showed intense reactivity (Figure 6). TTR genetic sequencing revealed a TTRV30M (p. TTRV50M) variant. ^99m^Tc-DPD scintigraphy, a noninvasive method for evaluation of ATTR cardiac amyloidosis, showed Perugini grade 3 (maximum). She was started on tafamidis as a disease-modifying therapy. Her sister was found to be an asymptomatic carrier by genetic testing.

ATTR autosomal dominant diseases are caused by the dissociation of the natively tetrameric protein transthyretin (TTR) and misfolding of the resulting monomers, which assembles into amyloid structures.^34^ TTR is mainly synthesized by the liver and is encoded on chromosome 18, with over 130 mutations in the *TTR* gene described. Outside Europe, Japan, and the United States, this form of amyloid is extensively reported in South China.^34^ Worldwide, V30M is considered the most frequent mutation. Misdiagnosis is common because of genetic and phenotypic heterogeneity, a wide age range of onset starting in the third decade, and variable family history (Table 2). Classically, the ATTR variant (ATTRv) phenotype manifests as neuropathy, with certain variants exclusively presenting with cardiomyopathy.^35^ Progression of CKD with heavy proteinuria and cardiac involvement influence long-term survival.

**Table 2:** Hereditary ATTR amyloidosis and variants with kidney involvement

Mutation Name (Including Signal Peptide)	Organ Involvement	Geographic Origin
Val30Leu (*p.Val50Leu*)	N, H, K	Japanese, American
Val30Met (*p.Val50Met*)	N, E, H, LM, K, CTS	European (Portuguese, Swedish), Japanese, American, Chinese
Phe33Cys (*p.Phe53Cys*)	CTS, E, K, H	American
Gly47Glu (*p.Gly67Glu*)	H, K, N	German, Italian
Ser52Pro (*p.Ser72Pro*)	N, H, K	British
Ser77Tyr (*p.Ser97Tyr*)	H, K, N	French, German, American
Tyr78Phe (*p.Tyr98Phe*)	N, H, K, L	Italian
Glu92Lys (*p.Val110Leu*)	H, L, K deposits, lung, GI	Japanese
Val94Ala (*p.Val114Ala*)	H, N, K	German, American

ATTR, transthyretin amyloid; CTS, carpal tunnel syndrome; E, eye; GI, gastrointestinal; H, heart; K, kidney; L, liver; LM, leptomeningeal; N, neuropathy.

Renal diagnosis is nuanced because deposits in the kidney do not consistently parallel the myelinated nerve fiber loss and may be missed. Medullary amyloidosis may predominate, with noticeable amyloid deposition in the absence of proteinuria. Without therapy, 10% of Portuguese patients with ATTRV30M progressed to stage 5 CKD and one third had pathological albuminuria.^36^ In a study from France, comprising 21 different *TTR* gene mutations, one third of patients developed CKD and 20.3% had proteinuria.^37^ In both studies, late onset (as in this case) was associated with CKD.^36,37^ In the United States, Renasight testing applied for clinical purposes demonstrated that 4.1% of patients have a pathogenic *TTR* gene mutation, all with a V122I (*p.Val142Ile*) variant.^38,39^

Wild-type TTR (ATTRwt) can also form amyloid fibrils with a structure surprisingly similar to patients with ATTRv, including V30M, P24S, and I84S. This suggests that not only fibril morphology, but also the conditions of fibril formation and deposition, are crucial for defining the phenotypic variability.^43^ ATTRwt causes cardiomyopathy in the elderly with cardiorenal syndrome and reduction in renal perfusion. In these patients, renal involvement is generally characterized by stage 2–3 CKD and absent or mild proteinuria.^36^ Similar long-term patterns occur in patients receiving liver transplant as the initial therapy. In ATTRv, cardiorenal treatment is limited by hypotension and hemodialysis may be an indication to treat refractory heart failure. Sodium-glucose cotransporter 2 inhibitor therapy is not an outlined practice in ATTR, and although small series describe good tolerance (in both ATTRwt and ATTRv), the supported efficacy requires extended follow-up.^41,42^

Treatment options for ATTR (Table 3) vary on the basis of organ involvement. Currently, the decreasing number of patients with CKD stage 4/5 is believed to be associated with antiproteinuric effects of the TTR stabilizer tafamidis and TTR silencing therapy patisiran.^42,43^ In mutations with kidney involvement (Table 2), regular assessment of urine albumin–creatinine ratio and kidney function are recommended, even in asymptomatic carriers.^44^ The disease-modifying therapies in ATTR amyloidosis should be maintained even in dialysis because the systemic disease will progress. None of the clinical trials included patients with CKD stage 5 patients, although there is no clear scientific basis for their exclusion. In addition, nonresponding proteinuric patients should switch therapy unless a neurological/cardiologic indication exists. Improving diagnostic technique and careful structuring of clinical trials will ultimately help long-term organ and patient survival.

**Table 3:** Treatment of ATTR amyloidosis: on clinical practice, under investigation and kidney impactful effects

Mechanism	Intervention		Indications (ATTRv and/or ATTRwt)	Kidney Effects/Prognosis
Substitution of the primary source of mutant TTR production	Transplantation	Organ		
		Liver	ATTRv The advance of pharmacological therapy made this indication rare	Post–liver transplantation cardiorenal syndrome in long-term survival patients because of progression of ATTR cardiomyopathy Chronic calcineurin inhibitor nephrotoxicity
		Liver and kidney	ATTRv Applied in CKD5 when neurological and heart conditions are favorable and no pharmacological therapy is approved	Complicated urinary tract infections due to neurogenic bladder Progression of cardiac amyloidosis and cardiorenal syndrome
		Liver and heart	ATTRv The advance of pharmacological therapy made this indication rare	Chronic calcineurin inhibitor nephrotoxicity
TTR stabilizers bind to the TTR homotetramers preventing dissociation into monomers	Tafamidis	Meglumine 20 mg	ATTRv Applied to neuropathy	Remission to normoalbuminuria in patients with UACR >300 mg/g and improved CKD staging^43^
		Meglumine 80 mg or tafamidis 61 mg	ATTRv or wt Approved for cardiomyopathy	The same findings as 20 mg dosage concerning CKD staging^45^
	Acoramidis	ATTRibute-CM Phase 3 trial 800 mg twice daily	ATTRv or wt cardiomyopathy Evaluation in progress	AKI occurred in 12.4% of patients on the drug versus 10.4% on the placebo (no significant difference in AKI)
	Diflunisal	250 mg twice daily	ATTRv or wt	CKD stage may worsen as it is a nonsteroidal anti-inflammatory drug
TTR silencers bind an degrade TTR mRNA block the synthesis of TTR^47^	Antisense oligonucleotides	Inotersen 284 mg subcutaneously once weekly	ATTRv In the United States, it will no longer be available; there is a transition plan to next-generation therapy (eplontersen)	Cases associated to AKI, crescentic GN, tubulointerstitial nephritis, low complement levels, PR3-ANCA positivity,^54^ and segmental and focal glomerulosclerosis^48^
		Eplontersen 45 mg, subcutaneous, once monthly	ATTRv neuropathy ATTRv or wt cardiomyopathy—evaluation in progress	Proteinuria 8%^49^
	Small interference RNA	Patisiran 0.3 mg/kg Every 3 wk	ATTRv neuropathy ATTRv or wt cardiomyopathy	Remission of albuminuria^37^
		Vutrisiran 25 mg every 3 mo	ATTRv neuropathy ATTRv or wt cardiomyopathy^50^	Absence of *de novo* kidney features
Permanently reduce or eliminate the production of abnormal TTR by directly targeting and editing the TTR gene in the liver	CRISPR/Cas9 gene editing^51^	NTLA-2001 Phase 3 trial	ATTRv or wt	Not described^a^
Clearance of transthyretin aggregates	IgG1 humanized mouse monoclonal antibody^52^	Coramitug (PRX004) Phase 2 trial	ATTRv or wt cardiomyopathy	Not described^a^
Depletion of ATTR fibrils through antibody-mediated phagocytosis	Recombinant human IgG1 monoclonal antibody^53^	ALXN2220 Phase 3 trial	ATTRv or wt cardiomyopathy	Not described^a^

ATTR, transthyretin amyloidosis; ATTRv, ATTR variant; ATTRwt, ATTR wild-type; CKD5, CKD stage 5; CRISPR/Cas9, clustered regularly interspaced palindromic repeats/Cas9; IgG1, immunoglobulin G subclass 1; TTR, transthyretin; UACR, urine albumin–creatinine ratio.

a The trial does not evaluate renal outcome; a trial specifically addressing renal outcomes is not available.

## Conclusion

Renal amyloidosis is an evolving area of diagnostic, clinical, and therapeutic research. Precise diagnosis of the type of amyloid is key to determining therapy and prognosis. Much has been described about AL and AA, but renal diagnosis of and therapies for ALECT2 and ATTR leave much to be desired. It is also important to appreciate the varied renal outcomes on the basis of the type of protein identified. In this article we reviewed select patients with renal amyloidosis and the associated clinical dilemmas. We also provided an update on the future therapeutic landscape.
